# Interaction between Alzheimer’s Disease and Cerebral Small Vessel Disease: A Review Focused on Neuroimaging Markers

**DOI:** 10.3390/ijms231810490

**Published:** 2022-09-10

**Authors:** Si Eun Kim, Hee Jin Kim, Hyemin Jang, Michael W. Weiner, Charles DeCarli, Duk L. Na, Sang Won Seo

**Affiliations:** 1 Department of Neurology, Samsung Medical Center, Sungkyunkwan University School of Medicine, Seoul 06351, Korea; 2 Neuroscience Center, Samsung Medical Center, Seoul 06351, Korea; 3 Samsung Alzheimer Research Center, Samsung Medical Center, Seoul 06351, Korea; 4 Department of Neurology, Inje University College of Medicine, Haeundae Paik Hospital, Busan 48108, Korea; 5 Center for Imaging of Neurodegenerative Diseases, University of California, San Francisco, CA 94121, USA; 6 Department of Neurology and Center for Neuroscience, University of California, Davis, CA 95616, USA; 7 Department of Health Sciences and Technology, Samsung Advanced Institute for Health Sciences and Technology (SAIHST), Sungkyunkwan University, Seoul 06355, Korea; 8 Stem Cell and Regenerative Medicine Institute, Samsung Medical Center, Seoul 06351, Korea; 9 Department of Clinical Research Design and Evaluation, SAIHST, Sungkyunkwan University, Seoul 06355, Korea; 10 Center for Clinical Epidemiology, Samsung Medical Center, Seoul 06351, Korea

**Keywords:** subcortical vascular cognitive impairment, Alzheimer’s disease, ß-Amyloid, tau, cerebral small vessel disease, interaction, positron emission tomography

## Abstract

Alzheimer’s disease (AD) is characterized by the presence of β-amyloid (Aβ) and tau, and subcortical vascular cognitive impairment (SVCI) is characterized by cerebral small vessel disease (CSVD). They are the most common causes of cognitive impairment in the elderly population. Concurrent CSVD burden is more commonly observed in AD-type dementia than in other neurodegenerative diseases. Recent developments in Aβ and tau positron emission tomography (PET) have enabled the investigation of the relationship between AD biomarkers and CSVD in vivo. In this review, we focus on the interaction between AD and CSVD markers and the clinical effects of these two markers based on molecular imaging studies. First, we cover the frequency of AD imaging markers, including Aβ and tau, in patients with SVCI. Second, we discuss the relationship between AD and CSVD markers and the potential distinct pathobiology of AD markers in SVCI compared to AD-type dementia. Next, we discuss the clinical effects of AD and CSVD markers in SVCI, and hemorrhagic markers in cerebral amyloid angiopathy. Finally, this review provides both the current challenges and future perspectives for SVCI.

## 1. Introduction

Dementia is a progressive and deteriorative syndrome that affects memory and other cognitive domains, which interferes with a daily living [1]. Alzheimer’s disease (AD) and vascular dementia (VaD) are the two most common causes of dementia in the elderly [2]. AD is characterized by senile plaques formed by β-amyloid (Aβ) and neurofibrillary tangles (NFTs) formed by hyperphosphorylated tau. These changes, along with loss of neurons, contribute to the symptoms of dementia [3]. Based on these core AD pathological features, including Aβ [A], tau [T], and neurodegeneration [N] biomarkers, the National Institute on Aging—Alzheimer’s Association (NIA-AA) proposed the AT(N) classification system [4]. A and T biomarkers are specific for the Aβ plaques and tau NFTs that constitute the hallmark neuropathologic signs of AD, respectively, while biomarkers of (N) (such as atrophy on magnetic resonance imaging, MRI) are not disease specific [4,5]. Brain atrophy is indicative of the considerable loss of neurons and synapses in the cerebral cortex [6]. Although the assessment of atrophy lacks specificity to determine whether the cause is cell loss or synaptic loss, cortical thickness is widely used as a surrogate marker for neuronal loss [7,8]. The NIA-AA research framework defines AD biologically based on neuropathologic change or biomarkers and considers cognitive impairment a symptom or sign of the disease rather than the definition of the disease [4].

Further, Alzheimer’s disease is frequently associated with other aging-related disorders such as cerebrovascular disease, Lewy body disease, transactive response DNA-binding protein of 43 kDa (TDP-43) proteinopathies, and argyrophilic grain disease [6]. AD pathology mixed with vascular disease is more frequent in the elderly population (also known as mixed pathology in dementia or mixed dementia) [9]. Vascular diseases include arteriolosclerosis, cerebral amyloid angiopathy (CAA), atherosclerosis, macroinfarcts, and microinfarcts [10]. In fact, previous studies have shown that AD combined with vascular disease is the most prevalent mixed pathology [9,11,12].

Vascular dementia is caused by ischemic or hemorrhagic brain lesions that are characterized by numerous clinical syndromes [13]. The most common forms of VaD in the elderly are subcortical vascular dementia (SVaD), strategic infarct dementia, and multi-infarct dementia [14]. VaD is generally known to be the second most common cause of dementia in later life among Caucasian populations, although it may be the most common cause in East Asia [15,16,17]. SVaD, one of the main forms of VaD, is characterized by extensive cerebral small vessel disease (CSVD), including white matter hyperintensities (WMHs) and multiple lacunes [18]. Vascular risk factors, such as age, hypertension, and diabetes mellitus, contribute to the development of CSVD MRI markers. These markers gradually form deposits in subcortical regions over several decades, eventually resulting in SVaD [19]. Thus, SVaD shows a progression pattern similar to that of AD, which reveals an insidious onset and gradual progression; however, it is dissimilar to that of multi-infarct dementia (another major form of vascular dementia). From this perspective, there is a prodromal state of SVaD, referred to as subcortical vascular mild cognitive impairment (svMCI). Subcortical vascular cognitive impairment (SVCI), which incorporates SVaD and svMCI, refers to cognitive impairment caused by subcortical vascular lesions [20,21,22,23,24].

AD-related cognitive impairment (ADCI) and SVCI are considered to lie on opposite ends of a single disease spectrum, where ADCI with non-ischemia lies at one end and SVCI without AD pathology lies at the other end [25]. One of the main reasons is that these two types of dementia share risk factors, such as age, hypertension, and diabetes [26]. In fact, these risk factors are known to be associated with AD-type dementia as well as SVCI [27]. The other reason is based on previous studies suggesting a strong association between AD and CSVD burden [28,29]. Several pathological studies have shown an overlap between AD and CSVD burden and their association with dementia. In particular, concurrent CSVD burdens are more commonly observed in AD-type dementia than in other neurodegenerative diseases [30]. AD dementia can develop in the presence of CSVD lesions [31]. Among patients with dementia, 38.0% (19/50) have AD and infarcts, 30.0% (15/50) have pure AD, and 12% (6/50) have vascular dementia [9]. The association between AD and CSVD could be explained by the possibility that CSVD hampers the clearance of Aβ [32,33,34].

In most dementia cases, lesions are pathologically identified after death. Therefore, we do not have exact information on the patients when they were at earlier stages. However, with advancements in molecular imaging, AD biomarkers have been detected in living AD patients at earlier stages of dementia. Abnormal levels of AD imaging markers can be quantified with specific positron emission tomography (PET) tracers, such as ^11^C-Pittsburgh Compound-B (PiB) [35], ^18^F-florbetapir [36], ^18^F-flutemetamol [37], and ^18^F-florbetaben [38] for Aβ, and ^18^F-flortaucipir (AV-1451) [39], ^18^FMK-6240 [40], ^18^F-PI-2620 [41], and ^18^F-RO-948 PET [42] for tau. Specifically, compared to cognitively normal individuals, patients with AD-type dementia show higher Aβ uptakes in the brain [35]. Furthermore, 20–30% of cognitively normal individuals and 40–60 % of individuals with mild cognitive impairment (MCI) both show Aβ positivity on PET [43,44]. According to a previous study, tau PET positivity in the temporal region has been shown to be 6.1% for cognitively normal individuals, 46.5% for MCI, and 88.6% for AD-type dementia [45]. Tracers for paired helical filament tau have also been reported to correspond to Braak’s pathological NFT stage and to be correlated with disease severity and symptom progression [46,47,48]. However, compared to AD, there has been relatively little interest in research using molecular imaging for SVCI.

In this review, we discuss the interaction between AD and CSVD biomarkers and the clinical effects of these two biomarkers using molecular imaging studies. More specifically, we discuss the following topics: (1) the frequency of AD imaging markers, including Aβ and tau in SVCI patients; (2) the relationship between AD markers and CSVD burdens; (3) potential distinct pathobiology of AD markers in SVCI compared to AD-type dementia; (4) the clinical effects of AD and CSVD markers in SVCI; (5) hemorrhagic markers in CAA and the clinical effects; and (6) current challenges and future perspectives.

## 2. Imaging Markers of Alzheimer’s Disease (AD) and Cerebral Small Vessel Disease (CSVD) in Subcortical Vascular Cognitive Impairment (SVCI)

### 2.1. Frequency of AD Imaging Markers in SVCI

AD markers are more commonly observed in patients with SVCI than in cognitively unimpaired individuals. Specifically, in svMCI patients, the frequencies of Aβ positivity have been reported to be about 30% [25,29,49]. SVaD patients tend to display more frequent Aβ positivity than svMCI patients, ranging from 30% to 53% [25,50,51]. In terms of the tau marker, it has been shown that tau positivity is 70% (14/20) in Aβ (+) ADCI patients, 25.9% (7/27) in Aβ (+) SVCI patients, and 6.1% (2/33) in Aβ (−) SVCI patients. [52].

### 2.2. Correlation between AD and CSVD Imaging Markers

**Figure 1:**
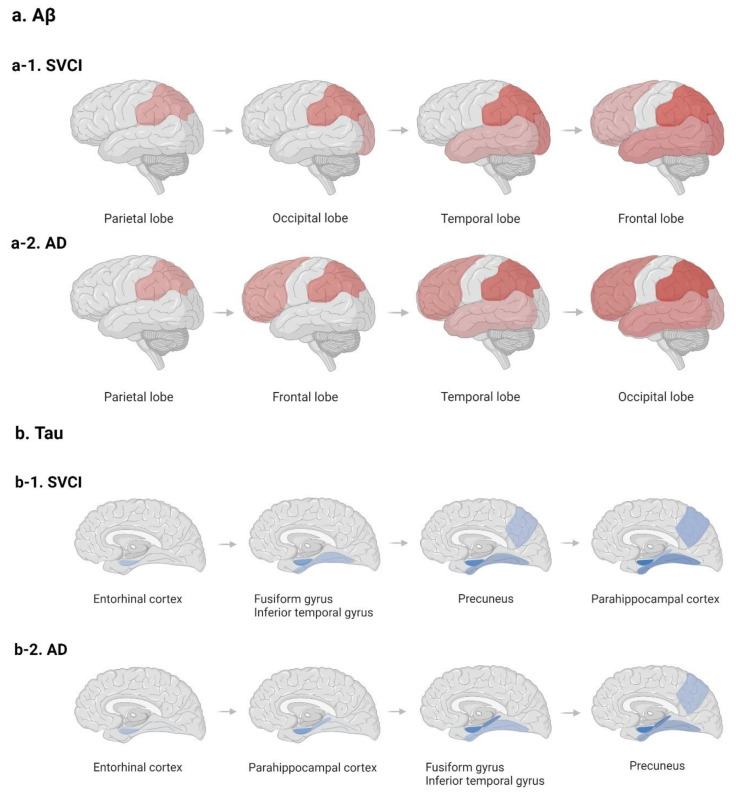
Spreading order of Aβ and tau in SVCI and AD, respectively. (**a**) Spreading pattern of Aβ in SVCI (**a-1**) and AD (**a-2**); (**b**) Spreading pattern of tau in SVCI (**b-1**) and AD (**b-2**). In the order of spreading Aβ in SVCI, unlike AD, Aβ accumulates in the occipital area before the temporal and frontal regions. In contrast to AD, tau accumulates in the fusiform gyrus and inferior temporal gyrus before the parahippocampal cortex in SVCI patients. Aβ—β-amyloid; SVCI—subcortical vascular cognitive impairment; AD—Alzheimer’s disease.

Molecular imaging studies have enabled us to investigate the relationship between AD markers and CSVD MRI markers throughout the whole brain. There is increasing evidence from these studies showing that AD marker uptake is correlated with WMH volume, which is a characteristic MRI marker of CSVD. This has been observed prominently in the posterior regions of the brain. In our previous study of 53 SVCI patients, a relationship between Aβ uptake and WMH volume was observed in *APOE4* non-carriers [53]. WMH volume is correlated with Aβ uptake in the posterior cerebral regions. Another study using clustering analyses classified SVCI patients and AD patients into the Aβ occipital-predominant and Aβ occipital-sparing groups. The frequency of the occipital-predominant group has been shown to be higher in SVCI patients (62.2%) than in AD patients (37.8%) [33]. Furthermore, the Aβ spreading pattern in patients with SVCI is quite different from in patients with ADCI. Specifically, the Aβ spreading pattern of patients with SVCI demonstrates that Aβ accumulates in the occipital area before the temporal and frontal regions, whereas in patients with ADCI, the parietal and fronto-temporal regions precede the occipital region. (Figure 1a) [33,54,55,56,57]. The predominant Aβ deposition in the occipital region, mainly observed in patients with SVCI, may be related to the distribution pattern of CAA or ischemic vulnerability of the posterior circulation [53]. CAA is primarily found in the occipital region [53,58]. Moreover, ischemic injury and dysfunction of the endothelial layer may lead to disruption of the blood–brain barrier (BBB), which in turn leads to the deposition of Aβ. Since the vertebrobasilar system, which is responsible for the posterior circulation, may be vulnerable to ischemia, SVCI patients may show Aβ deposition primarily in the posterior region [53]. Figure 1a illustrates the spreading pattern of Aβ in AD, compared with that in SVCI. Interestingly, the Aβ spreading pattern in patients with ADCI developed using molecular imaging evidence seems to be different from that based on pathological studies. That is, a pathologic study conducted by Braak and Braak showed an early pattern of Aβ deposits in the basal parts of the frontal, temporal, and occipital lobes (Stage A) [46,59]. However, several molecular imaging studies suggest that there are diverse early Aβ accumulating regions such as the precuneus, posterior cingulate, isthmus cingulate, insula, and medial and lateral orbitofrontal cortices, in which several of the core regions of the default mode network are located [60,61,62].

In terms of the relationship between CSVD and tau, previous studies have suggested that ischemia might increase tau burdens regardless of the amyloid pathway [63]. Animal studies have also shown an association between increased cerebrovascular pathology and tau formation [64]. In vivo imaging studies have shown that CSVD burden may be associated with higher tau accumulation in the inferior temporal regions regardless of Aβ positivity [65]. Furthermore, in terms of tau spreading order, patients with SVCI are quite different from patients with ADCI. Unlike in ADCI, tau accumulates earlier in the fusiform gyrus and inferior temporal gyrus than in the parahippocampal cortex in SVCI (Figure 1b) [65,66].

### 2.3. Potential Distinct Pathobiology of AD Markers in SVCI

**Figure 2:**
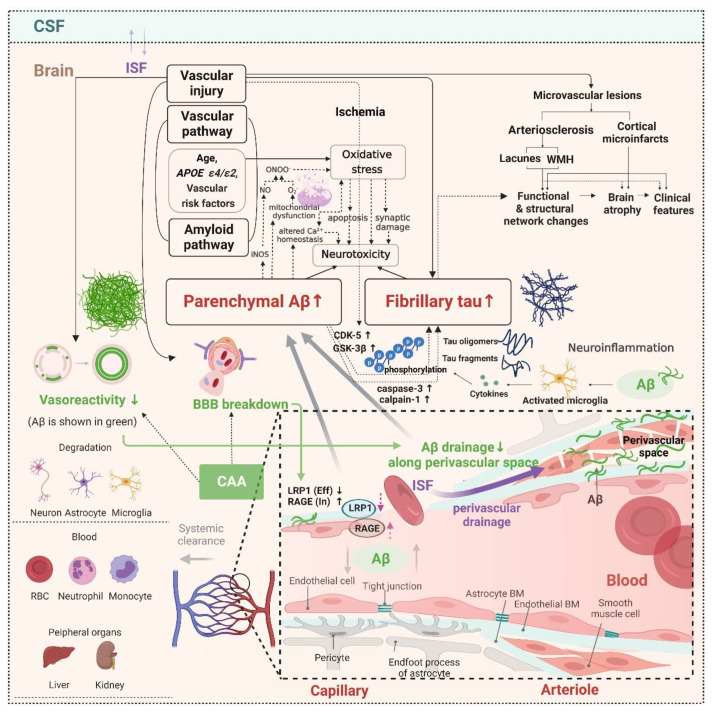
Potential mechanism of Aβ and tau deposition in SVCI. The CSVD burden is associated with Aβ and tau deposition in SVCI. Ischemic events can lead to Aβ deposition by reducing Aβ clearance via BBB breakdown or deficits in perivascular drainage of Aβ from the brain interstitial fluid. BBB breakdown causes faulty transport of Aβ by reducing LRP1 levels and increasing RAGE levels, resulting in impaired clearance of toxic Aβ species. Aβ accelerates the tau hyperphosphorylation by mediating the activation of protein kinases, including CDK-5 and GSK-3β. In addition, Aβ induces the activation of caspase-3 and calpain-1, and the cleavage of tau generates neurotoxic tau fragments. The association between Aβ and tau aggregation may involve microglial activation. Soluble Aβ oligomers have been known to activate microglial cells. Microglial activation precedes tau aggregation and facilitates tau hyperphosphorylation through cytokine release and the subsequent NFT formation. Vascular risk factors can also induce tau accumulation. Ischemia caused by vascular injury may activate CDK-5 and GSK-3 β, resulting in tau phosphorylation. Moreover, vascular risk factors and the accumulation of Aβ plaques lead to oxidative stress. Oxidative stress may also be caused by several mechanisms, such as mitochondrial dysfunction or inflammatory responses. It may manifest as damage to synapses and changes in Ca^2+^ homeostasis, resulting in an apoptotic cascade and neurotoxicity. CSVD—cerebral small vessel disease; Aβ—β-amyloid; BBB—blood–brain barrier; LRP1—low-density lipoprotein receptor-related protein 1; RAGE—receptor for advanced glycosylation end products; CDK-5—cyclin-dependent kinase 5; GSK-3β—glycogen synthase kinase 3β; NFTs—neurofibrillary tangles; ISF—interstitial fluid; CSF—cerebrospinal fluid; WMH—white matter hyperintensity; BM—basement membrane.

Considering that SVCI and ADCI patients show different spreading patterns of AD imaging markers, there may be differences in the potential pathobiology of AD biomarkers between SVCI and ADCI patients. In patients with SVCI, vascular risk factors may lead to Aβ deposition. Several cohort studies have reported an association between vascular risk factors and Aβ deposition (Table 1) [67,68,69,70,71,72]. This Aβ deposition is increased by impaired Aβ clearance via a deficit in perivascular drainage of Aβ and breakdown of the BBB (Figure 2) [73,74]. BBB breakdown causes faulty transport of Aβ through reduced levels of low-density lipoprotein receptor-related protein 1 (LRP1) and increased levels of receptor for advanced glycation end products (RAGE). These changes eventually lead to impaired clearance of toxic Aβ species [75,76]. Furthermore, Aβ accelerates the tau hyperphosphorylation by mediating the activation of protein kinases, including cyclin-dependent kinase 5 (CDK-5) and glycogen synthase kinase 3β (GSK-3β) [77,78]. In addition, Aβ induces the activation of caspase-3 and calpain-1 and the cleavage of tau, generating neurotoxic tau fragments (Figure 2) [79,80]. The link between Aβ and tau aggregation may involve microglial activation [81]. Soluble Aβ oligomers are known to activate microglial cells [82]. Mouse studies on transgenic AD have revealed that the microglial activation precedes tau aggregation [83] and facilitates tau hyperphosphorylation through cytokine release with subsequent NFT formation [84]. There are two potential mechanisms that may explain how vascular risk factors induce tau accumulation. One hypothesis is that ischemia may activate CDK-5 and GSK-3β, resulting in tau phosphorylation [85]. Activation of CDK-5 occurs when ischemia inhibits the pumping of calcium ions out of cells and raises intracellular calcium levels [86,87]. GSK-3β is activated by ischemia through decreased activity of the phosphatidylinositol 3-kinase/Akt pathway [88,89]. Moreover, vascular risk factors and the accumulation of Aβ plaques lead to oxidative stress [90,91,92]. Oxidative stress may also be caused by several mechanisms, such as mitochondrial dysfunction or inflammatory responses [92]. It may manifest as damage to synapses and changes in Ca^2+^ homeostasis, resulting in an apoptotic cascade and neurotoxicity [92] (Figure 2).

Notably, there are distinct effects of *APOE* genotyping on Aβ deposition between patients with SVCI and ADCI. Specifically, apolipoprotein E4 (*APOE4*) is a risk factor for Aβ positivity in patients with ADCI and SVCI. Apolipoprotein E2 (*APOE2*) is a protective factor in ADCI (OR = 0.43); however, it is a risk factor in SVCI (OR = 2.26) [93]. Thus, *APOE2* might accelerate apolipoprotein E leakage in the vessel walls of patients with SVCI, which in turn leads to impaired vascular drainage of Aβ. This impaired drainage eventually results in increased Aβ burdens in the brain parenchyma [93]. Alternatively, *APOE2* may contribute to the development of CAA, which in turn leads to increased CSVD [93].

### 2.4. Clinical Effects of AD and CSVD Markers in SVCI Patients

There has been some debate related to the clinical effects of Aβ and CSVD imaging markers. In fact, among patients with extensive WMHs, some tend to show severe dementia symptoms, while others have no symptoms. In this regard, our previous studies investigated which imaging markers might affect the clinical features of SVCI and found that AD biomarkers and CSVD independently affect cognition, abnormal behavior, and gait disturbances [29,32,51,65,94,95,96]. A cross-sectional study has reported that Aβ uptake is only associated with memory dysfunction, whereas CSVD burden is associated with memory, visuospatial, and frontal executive functions [94]. Longitudinal cohort studies have also shown that Aβ positivity is associated with faster cognitive decline in patients with SVaD [51] and higher conversion to dementia in patients with svMCI [32]. In terms of abnormal behavior, Aβ predicts the signs of delusions and irritability, while CSVD burdens are associated with other behavioral symptoms, such as apathy and depression [97]. In addition, periventricular WMHs are the most important predictor of gait disturbances [98].

SVCI patients show distinct brain structural and cognitive trajectories based on AT (Aβ/tau) biomarker profiles [52]. A previous study showed that patients in the A+T+ group predicted a more rapid decline in structural and cognitive trajectories than those in the A+T− group, followed by those in the A−T− group [52]. Moreover, AD markers and CSVD burden have a synergistic effect on cognitive decline. In a cross-sectional study, significant interactions between WMHs and Aβ uptake were apparent in visuospatial function, suggesting that CSVD and Aβ synergistically affect cognitive impairment [29]. A longitudinal study comparing patients with SVCI and ADCI who had similar tau levels has shown that as Aβ turns positive, SVCI shows a steeper cognitive decline compared to the ADCI group [99]. In addition, as tau levels increase, the SVCI group shows a steeper cognitive decline than the ADCI group [99]. These findings indicate that there are interactive effects between AD markers and CSVD on cognitive decline.

**Figure 3:**
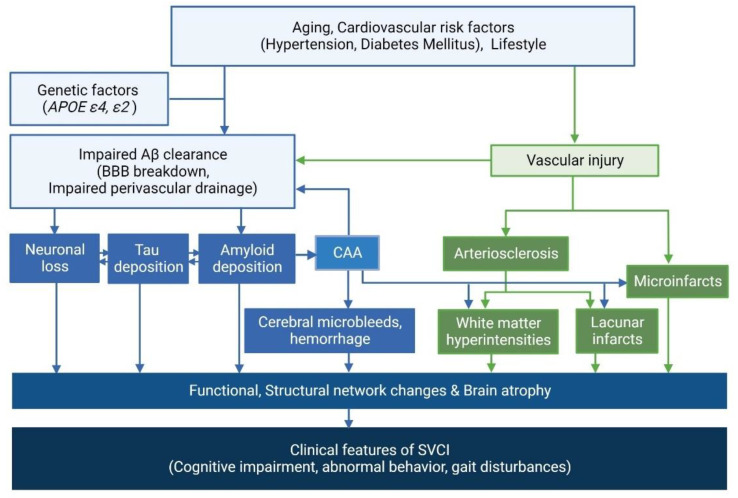
Mechanisms of SVCI. SVCI is caused by various factors, including increasing age, diabetes mellitus, hypertension, genetic predisposition such as *APOE ε4* and *ε2,* and arteriosclerosis. This can lead to Aβ deposition by impairing Aβ clearance. SVCI is also related to widespread white matter hyperintensities or multiple lacunar infarctions, which have been gradually deposited in subcortical regions for several decades. CAA is another factor known to be associated with SVCI. SVCI—subcortical vascular cognitive impairment; *APOE*—apolipoprotein E; Aβ—β-amyloid; BBB—blood–brain barrier; CAA—cerebral amyloid angiopathy.

Furthermore, Aβ and CSVD affect specific downstream imaging markers, such as network changes and brain atrophy in specific regions, which in turn lead to the development of these corresponding clinical outcomes [32,95,100]. Specifically, Aβ uptake is associated with cortical thinning in the medial temporal regions including hippocampal changes, which in turn leads to memory dysfunction. In contrast, CSVD burdens are primarily associated with frontal thinning [101] and white matter network disruption [95], which in turn leads to frontal dysfunction. In addition, a three-year longitudinal study has shown that time-varying Aβ and CSVD affects the temporoparietal and frontal thinning, respectively, which in turn contributes to the corresponding cognitive decline [32]. Another cross-sectional study has demonstrated that Aβ positivity and CSVD severity are independently associated with higher tau uptake in the medial and inferior temporal regions, respectively [65]. Moreover, increased tau uptake can mediate the relationship between Aβ and CSVD uptake and cognitive impairment, indicating that tau is another important common downstream marker of Aβ and CSVD burdens. The overall mechanisms of SVCI are summarized in Figure 3.

### 2.5. Hemorrhagic Markers in Cerebral Amyloid Angiopathy (CAA) and the Clinical Effects

CAA is characterized by Aβ deposition in the small arteries of the meninges and cortex, which causes vascular dysfunction and brain injury [102]. CAA is clinically and radiologically characterized by lobar intracerebral hemorrhage (ICH), strictly lobar cerebral microbleeds (CMBs) and cortical superficial siderosis (CSS) [103]. CAA is generally related to Aβ parenchymal aggregates, such as neuritic and diffuse plaques, although it can also occur pathologically without evident AD neuropathological changes [104].

Generally, the anatomical location of CMBs reflects their underlying etiology. Specifically, deep CMBs are presumed to be due to hypertensive CSVD, whereas lobar CMBs may reflect CAA [105]. In a cross-sectional study, consistent with previous studies, Aβ uptake has been shown to be associated with lobar CMBs [23]. CSVD is also associated with lobar CMBs as well as deep CMBs [23]. Aβ uptake and CSVD synergistically affect the development of lobar CMB [23]. Furthermore, a longitudinal study demonstrated that longitudinal measures of Aβ uptake and lacunes synergistically affect the development of lobar CMBs [69]. According to Thal’s CAA pathologic stage, CAA pathology extends sequentially from leptomeningeal and cortical vessels to cerebellar vessels and eventually to the striatum and brainstem vessels [106]. Patients with both cerebellar and lobar CMBs are more likely to present with CAA features, whereas deep CMBs, regardless of the presence of both lobar and cerebellar CMBs, are more likely to represent underlying hypertensive angiopathy than CAA features [107]. Interestingly, restricted superficial cerebellar CMBs refer to CAA imaging markers, whereas the involvement of the cerebellar dentate nucleus might be equivalent to deep CMB [107].

Our previous study showed that the frequency of *APOE4* was higher in Aβ (+) CAA than in Aβ (−) CAA, whereas *APOE2* was associated with overt hemorrhagic markers of CAA, such as lobar ICH and CSS [108]. These findings are consistent with other studies showing that *APOE4* is related to the deposition of Aβ burdens [109,110], and *APOE 2* is related to the breakdown of blood vessel walls [111]. In addition, the number of lobar CMB and the presence of CSS can predict Aβ (+), whereas ischemic CSVD markers can predict Aβ (−) [108].

A previous study investigating the clinical effects of CAA hemorrhagic markers has shown that multiple lobar CMBs are related to cortical thinning across all cortical regions, and that CSS is associated with frontal thinning, which in turn contributes to the corresponding cognitive decline [112]. Furthermore, path analyses have shown that the relationships between CAA hemorrhagic markers and cognitive impairments are partially mediated by thinning in cortical regions related to specific cognitive impairments [112]. A previous study investigated the clinical outcomes of parenchymal Aβ in patients with CAA, and showed that Aβ (+) CAA shows a steeper decline in multiple cognitive domains (including language, visuospatial, memory, and frontal dysfunctions) than Aβ (−) CAA [108].

## 3. Current Challenges and Future Perspectives

In SVCI, numerous potential biomarkers have been discovered using neuroimaging techniques, which were the focus of this review, as well as neuropathological research or genetic testing. These can be grouped broadly into the following categories: clinical biomarkers (neurobehavioral assessment); neuroimaging biomarkers, including WMHs and lacunes; biochemical biomarkers (serum, plasma, and CSF biomarkers); pathological biomarkers; and genetic biomarkers [113,114]. However, due to the lack of specific biomarkers for SVCI, additional extensive research on new biomarkers is necessary. Furthermore, the expansion of the AT(N) system to an ATV(N) framework is recommended [115,116]. Adopting vascular imaging biomarkers will improve the depth and accuracy of biomarker characterization in people along the AD continuum [115,116,117,118].

Research on dementia could further advance by recognizing and incorporating abundant knowledge on therapies to modulate vascular dysfunction and how to prevent and treat vascular illnesses. Treatment strategies for SVCI include slowing the progression of CSVD and improving clinical symptoms. For example, the modification of vascular risk factors, including hypertension and diabetes, has been recommended for the prevention of AD and SVCI [119,120,121,122,123]. Additionally, studying the pathways associated with Aβ deposition in SVCI may offer potential targets for treatment. For instance, strategies include cell-based therapies, which aim to promote the clearance of Aβ through the perivascular drainage pathway and BBB, such as upregulation of LRP1 and blockage of RAGE [124,125,126,127,128]. These strategies may provide important therapeutic applications that prevent the buildup of Aβ in the brain and protect vessels against damage in SVCI.

## 4. Conclusions

This review highlights the relationship between AD and SVCI. We focused on the interactions between AD and CSVD markers, potential distinct pathobiology, and clinical effects, based on molecular imaging studies. Therapeutic strategies are needed based on an understanding of the interactions between AD and CSVD markers in SVCI.

## Figures and Tables

**Table 1:** Cohort studies investigating the association between vascular risk factors and brain β-amyloid deposition.

Study (Country)	Length of the Study	Number of Study Participants (Age, Mean [SD])	Vascular Risk Factors	Measurement of Brain β-Amyloid Load	Results
Gottesman et al. (2017) (USA) [67]	Evaluation of vascular risk factors since 1987–1989 with ^18^F-florbetapir PET scans in 2011–2013	322 without dementia (27% MCI) (75.8 [5.3])	HTN, DM, BMI ≥ 30, TC ≥ 200 mg/dL, current smoking status	^18^F-florbetapir PET (SUVR)	(1) Association between elevated BMI in midlife and elevated SUVR (OR: 2.06, 95% CI: 1.16–3.65) (2) OR for elevated SUVR and 1 vascular risk factor: 1.88 (95% CI: 0.95–3.72), OR for elevated SUVR and 2 or more vascular risk factors: 2.88 (95% CI: 1.46–5.69)
Hughes et al. (2018) (USA) [68]	Evaluation of vascular risk factors since 1987–1989 with ^18^F-florbetapir PET scans in 2011–2013	321 (27% MCI) (76 [5])	Arterial stiffness by pulse wave velocity (PWV, carotid-femoral [cfPWV] and heart-carotid [hcPWV])	^18^F-florbetapir PET (SUVR)	(1) Association between greater central stiffness (hcPWV) and greater Aβ deposition (OR: 1.31, 95% CI: 1.01–1.7) (2) Association between cfPWV and a higher odds of Aβ-positive scans (OR: 1.4, 95% CI: 1.1–2.1).
Rabin et al. (2018) (USA) [69]	7 years	223 clinically normal older adults (73.7 [6.0])	Framingham Heart Study general cardiovascular disease (FHS-CVD) risk score (age, sex, antihypertensive treatment, SBP, BMI, history of DM, and current cigarette smoking status)	^11^C-PiB PET (DVR)	(1) Associations of a higher FHS-CVD risk score (β = −0.064; −0.094 to −0.033; *p* < 0.001) and higher Aβ burden (β = −0.058; −0.079 to −0.037; *p* < 0.001) with faster cognitive decline (2) Synergistic effect of FHS-CVD risk factors and Aβ burden (β = −0.040, 95% CI: −0.062 to −0.018; *p* < 0.001)
Arfanakis et al. (2020) (USA) [70]	25 years	603 (No cognitive impairment: 178, MCI: 154, dementia: 271) (age at death: 90 [7]; No cognitive impairment: 88 [7], MCI: 90 [6], dementia: 90 [7])	HTN, DM, smoking, history of heart disease	Neuropathologic examination	Association between WMH burden and both vascular and Alzheimer’s pathologies (arteriolosclerosis (*p* < 10^−4^), gross (*p* < 10^−4^) and microscopic infarcts (*p* = 0.04), Aβ plaques (*p* = 0.028)
Kobe et al.(2020) (Canada) [71]	7 years	215 participants (PREVENT-AD cohort of cognitively unimpaired individuals) (62.3 [5.0])	TC, HDL, LDL cholesterol levels, SBP, DBP, pulse pressure, Framingham Coronary Risk Profile (age, sex, SBP, DBP, HDL, LDL, smoking, DM)	^18^F-NAV 4694 PET (SUVR)	Association of vascular risk factors with Aβ burden but not tau burden (only among individuals who were not using vascular medications) TC level (β = −0.002 [SE, 0.001]; *p* = 0.02), LDL cholesterol level (β = −0.002 [SE,0.001]; *p* = 0.006), SBP (β = −0.006 [SE, 0.002]; *p* = 0.02), pulse pressure (β = −0.007 [SE, 0.002]; *p* = 0.004), and Framingham Coronary Risk Profile score (β = −0.038 [SE, 0.011]; *p* = 0.001)
Lockhart et al. (2022) (USA) [72]	19 years (enrollment, 2000–2002; 1^st^ cognitive abilities screening, 2010–2012; 2nd screening, 2016–2018)	159 participants (49.7% African-American, 50.3% White) (baseline age 55.8 [6.7])	FSRP, CAIDE, ASCVD (All vascular risk factor scores include age, sex, SBP); FSRP, ASCVD (DM, antihypertensive treatment, smoking); CAIDE, ASCVD (TC)	^11^C-PiB PET (SUVR)	Association of higher baseline Framingham stroke risk profile (FSRP) (*p* = 0.014) and Cardiovascular Risk Factors, Aging, and Incidence of Dementia (CAIDE) scores (*p* = 0.004) with global brain Aβ

Abbreviations: Standard Deviation—SD; MCI—mild cognitive impairment; HTN—hypertension; DM—diabetes mellitus; BMI—body mass index; TC—total cholesterol; LDL—low-density lipoprotein; SUVR—standardized uptake value ratio; OR—odds ratio; SBP—systolic blood pressure; DBP—diastolic blood pressure; Aβ—β-amyloid; DVR—distribution volume ratio; WMH—white matter hyperintensity; PREVENT-AD—Presymptomatic Evaluation of Experimental or Novel Treatments for Alzheimer Disease; FSRP—Framingham stroke risk profile; CAIDE—Cardiovascular risk factors, aging and incidence of dementia risk score; ASCVD—Atherosclerotic cardiovascular disease risk estimate from the pooled cohort equation.

## Data Availability

Not applicable.
